# Complete chloroplast genome sequences of the medicinal plant *Aconitum transsectum* (Ranunculaceae): comparative analysis and phylogenetic relationships

**DOI:** 10.1186/s12864-023-09180-0

**Published:** 2023-02-28

**Authors:** Niu Yanfei, Su Tai, Wu Chunhua, Deng Jia, Yang Fazhong

**Affiliations:** 1 Key Laboratory for Forest Resources Conservation and Utilization, Southwest Mountains of China, Southwest Forestry University, Kunming, 650224 China; 2 Yunnan Institute of Materia Medica, Kunming, 650111 China; 3 Yunnan Baiyao Group Chinese Medicinal Resources Division, Kunming, 650500 China; 4 Key Laboratory of State Forestry and Grassland Administration On Highly-Efficient Utilization of Forestry Biomass Resources in Southwest China, Kunming, 650224 China; 5 College of Chemical Engineering, Southwest Forestry University, Kunming, 650224 China

**Keywords:** *Aconitum transsectum*, Sequencing, Chloroplast genome, SSR, Phylogenetic analysis

## Abstract

**Background:**

*Aconitum transsectum* Diels. (Ranunculaceae) is an important medicinal plant that is widely used in traditional Chinese medicine, but its morphological traits make it difficult to recognize from other *Aconitum* species. No research has sequenced the chloroplast genome of *A.transsectum*, despite the fact that phylogenetic analysis based on chloroplast genome sequences provides essential evidence for plant classification*.*

**Results:**

In this study, the chloroplast (cp) genome of *A. transsectum* was sequenced, assembled, and annotated. *A. transsectum* cp genome is a 155,872 bp tetrameric structure including a large single copy (LSC, 87,671 bp) and a small single copy (SSC, 18,891 bp) section, as well as a pair of inverted repeat sequences (IRa and IRb, 25,894 bp each). 131 genes are encoded by the complete cp genome, comprising 86 protein-coding genes, 37 tRNAs, and 8 rRNAs. The most favored codon in the *A. transsectum* cp genome is AUG, and 46 repeats and 241 SSRs were also identified. The *A. transsectum* cp genome is similar in size, gene composition, and IR expansion and contraction to the cp genomes of seven Ranunculaceae species. Phylogenetic analysis of cp genomes of 28 plants from the Ranunculaceae family shows that *A. transsectum* is most closely related to *A. vilmorinianum*, *A*. *episcopale*, and *A. forrestii* of Subgen. *Aconitum*.

**Conclusions:**

Overall, this study provides complete cp genome resources for *A. transsectum* that will be beneficial for identifying potential.

**Supplementary Information:**

The online version contains supplementary material available at 10.1186/s12864-023-09180-0.

## Background

*Aconitum transsectum* Diels. is a species of *Aconitum* in the Ranunculaceae family, mainly distributed in northwestern Yunnan, the roots are highly toxic and have medicinal value, and Chinese folk used them to treat rheumatism and other diseases [1]. Genus *Aconitum* belongs to the Ranunculaceae family, which has over 350 species worldwide and is found primarily in temperate parts of the Northern Hemisphere, particularly Asia, Europe, and North America [2]. China has recorded more than 200 species, the most abundant plant resources of the genus in the country [3]. The genus *Aconitum* is widely distributed in Taiwan and the mainland provinces of China except for Hainan provinces, mostly in the alpine zone of northern Yunnan, western Sichuan, and eastern Tibet, followed by a number of species in the northeastern provinces [1]. The genus *Aconitum* is a traditional Chinese medicinal plant with over 2000 years of use, it is classified into three subgenera: *Aconitum*, *Gymnaconitum*, and *Paraconitum*, with about 36 species available for medical use in China [4]. Genus *Aconitum* is widely used in traditional Chinese medicine, but it is not easy to distinguish among the species in terms of morphological characteristics, and there are huge differences in chemical composition, which makes it easy to endanger the lives of the wrong species during the actual use of medicine [5].

With the decrease in the cost of chloroplast whole-genome sequencing and the maturity of data analysis technology in latest years, a growing number of scholars have conducted chloroplast whole-genome studies, and chloroplast whole-genome data analysis has gradually become an efficient tool for species identification and species evolution studies [6]. Chloroplast (cp), a unique organelle of green plants and algae, is the location of photosynthesis in plants [7], and has its own genetic system consisting of a closed loop of double-stranded DNA molecules. The cp not only have their own genetic material, but are also a relatively independent genetic system capable of semi-autonomous replication under conditions where the nucleus provides genetic information [8]. Higher plants' cp genomes often have a tetrameric structure with a large single copy (LSC), a small single copy (SSC), and two inverted repeats (IRa and IRb) [9]. The cp genomes are small and highly conserved in sequence and structure, making them well suited for phylogenetic studies of complex plant populations [10–12].

It is the first time that the entire cp genome of *A. transsectum* was sequenced and analyzed, and the cp genome differences between *A. transsectum* and other related species were evaluated in this study. Based on cp genomic data, a phylogenetic tree of 26 *Aconitum* species and 2 *Delphinium* species was constructed to investigate the affinities between *A. transsectum* and other species, as well as to provide a theoretical foundation for understanding *A. transsectum*'s cp genomic characteristics and phylogenetic relationships.

## Result

### Chloroplast genome characterization

**Fig. 1:**
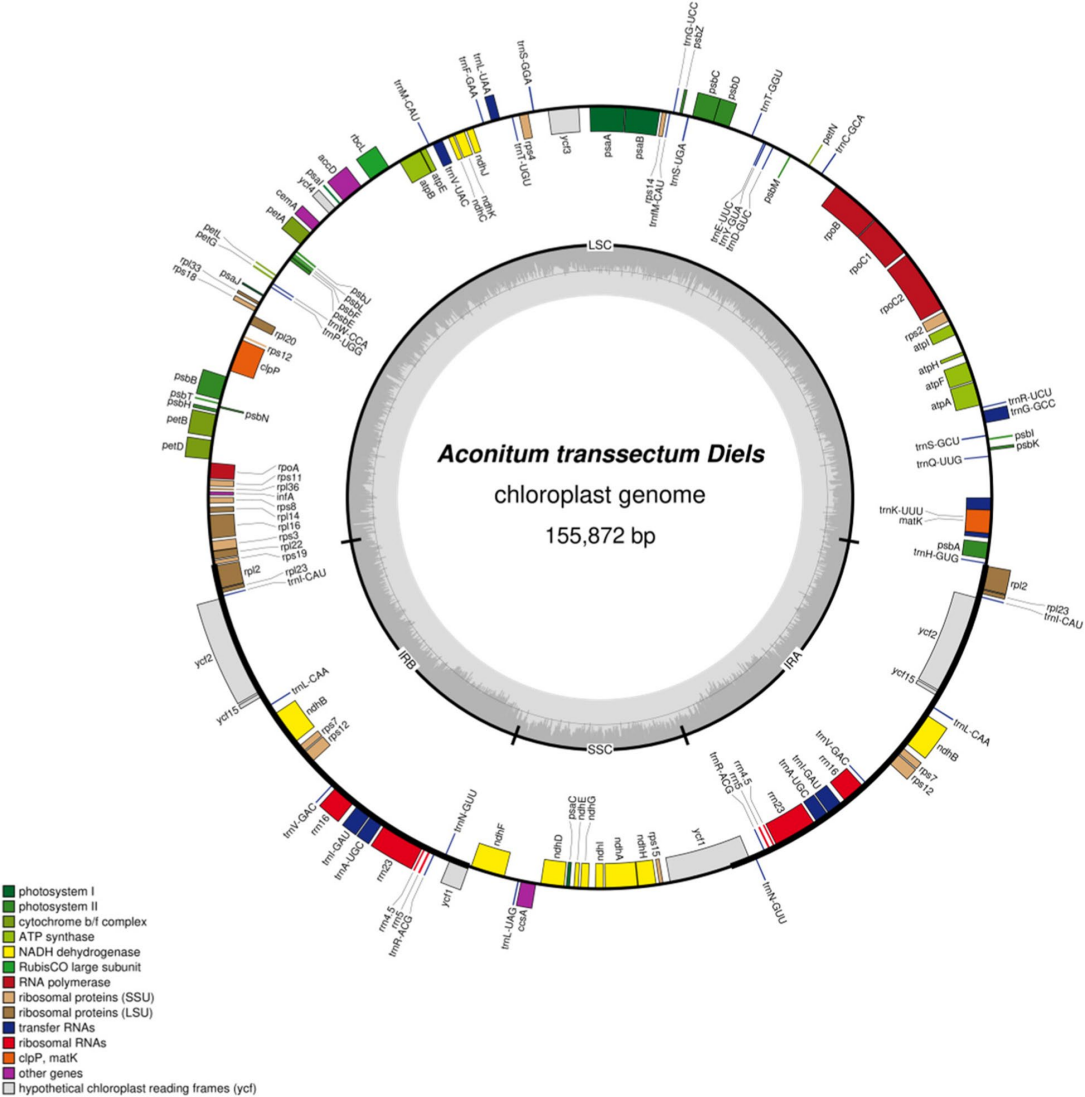
Gene map of *A. transsectum* cp genome

The cp genome of *A. transsectum* was 155,872 bp with a standard cyclic quadripartite structure, containing a couple of IR regions IRa and IRb (25,894 bp), an 18,891 bp SSC region and an 87,671 bp LSC region (Table 1 and Fig. 1). Overall GC content of the cp genome was 38.07%, while GC content was unevenly distributed across the cp genome, the IR region (42.97%) had a higher GC content than the LSC (36.19%) and SSC (32.54%) regions.

**Table 1:** The Characteristics of *A. transsectum* cp genome

Category	Item	Describe
Cp genome structure	Cp genome/bp	155,872
	LSC/bp	87,671
	SSC/bp	18,891
	IRa/IRb/bp	25,894
Gene composition	Cp gene	131
	tRNA	37
	rRNA	8
	mRNA	86
	pseudo	0
GC Content (%)	Cp gene	38.07%
	LSC	36.19%
	SSC	32.54%
	IRa/IRb	42.97%

The cp genome of *A. transsectum* has 131 predicted functional genes, including 86 protein-coding genes, 37 tRNA genes, and 8 rRNA genes, with no pseudogenes (Table 2). In the IR regions of the cp genomes, there were 19 duplicated genes, including 4 rRNA genes (rrn16, rrn23, rrn4.5, and rrn5), 8 protein-coding genes (ndhB, rpl2, rpl23, rps12, rps7, ycf15, ycf1 and ycf2), and 7 tRNA genes (trnL-CAA, trnI-CAU, trnI-GAU, trnN-GUU, trnA-UGC, trnR-ACG, trnV-GAC). In addition, among these 131 genes, 14 genes (atpF, ndhA, ndhB, petB, petD, rpl2, rpl16, rpoC1, trnI-GAU, trnG-GCC, trnL-UAA, trnV-UAC trnA-UGC, trnK-UUU,) contained one intron and 3 genes (rps12, ycf3, clpP) contained two introns.

**Table 2:** Genes in cp genome of *A. transsectum*

Category	Gene group	Gene name
Photosynthesis	Subunits of photosystem I	psaA, psaB, psaC, psaI, psaJ
	Subunits of photosystem II	psbA, psbB, psbC, psbD, psbE, psbF, psbH, psbI, psbJ, psbK, psbL, psbM, psbN, psbT, psbZ
	Subunits of NADH dehydrogenase	ndhA^*^, ndhB^*^(2), ndhC, ndhD, ndhE, ndhF, ndhG, ndhH, ndhI, ndhJ, ndhK
	Subunits of cytochrome b/f complex	petA, petB^*^, petD^*^, petG, petL, petN
	Subunits of ATP synthase	atpA, atpB, atpE, atpF^*^, atpH, atpI
	Large subunit of rubisco	rbcL
	Subunits photochlorophyllide reductase	-
Self-replication	Proteins of large ribosomal subunit	rpl14, rpl16^*^, rpl2^*^(2), rpl20, rpl22, rpl23(2), rpl33, rpl36
	Proteins of small ribosomal subunit	rps11, rps12^**^(2), rps14, rps15, rps18, rps19, rps2, rps3, rps4, rps7(2), rps8
	Subunits of RNA polymerase	rpoA, rpoB, rpoC1^*^, rpoC2
	Ribosomal RNAs	rrn16(2), rrn23(2), rrn4.5(2), rrn5(2)
	Transfer RNAs	trnA-UGC^*^(2), trnC-GCA, trnD-GUC, trnE-UUC, trnF-GAA, trnG-GCC^*^, trnG-UCC, trnH-GUG, trnI-CAU(2), trnI-GAU^*^(2), trnK-UUU^*^, trnL-CAA(2), trnL-UAA^*^, trnL-UAG, trnM-CAU, trnN-GUU(2), trnP-UGG, trnQ-UUG, trnR-ACG(2), trnR-UCU, trnS-GCU, trnS-GGA, trnS-UGA, trnT-GGU, trnT-UGU, trnV-GAC(2), trnV-UAC^*^, trnW-CCA, trnY-GUA, trnfM-CAU
Other genes	Maturase	matK
	Protease	clpP^**^
	Envelope membrane protein	cemA
	Acetyl-CoA carboxylase	accD
	c-type cytochrome synthesis gene	ccsA
	Translation initiation factor	infA
	other	-
Genes of unknown function	Conserved hypothetical chloroplast ORF	ycf1(2), ycf15(2), ycf2(2), ycf3^**^, ycf4

Gene^*^: Gene with one intron; Gene^**^: Gene with two introns; Gene (2): Genes duplicated in the IR regions

### Codon usage bias

**Fig. 2:**
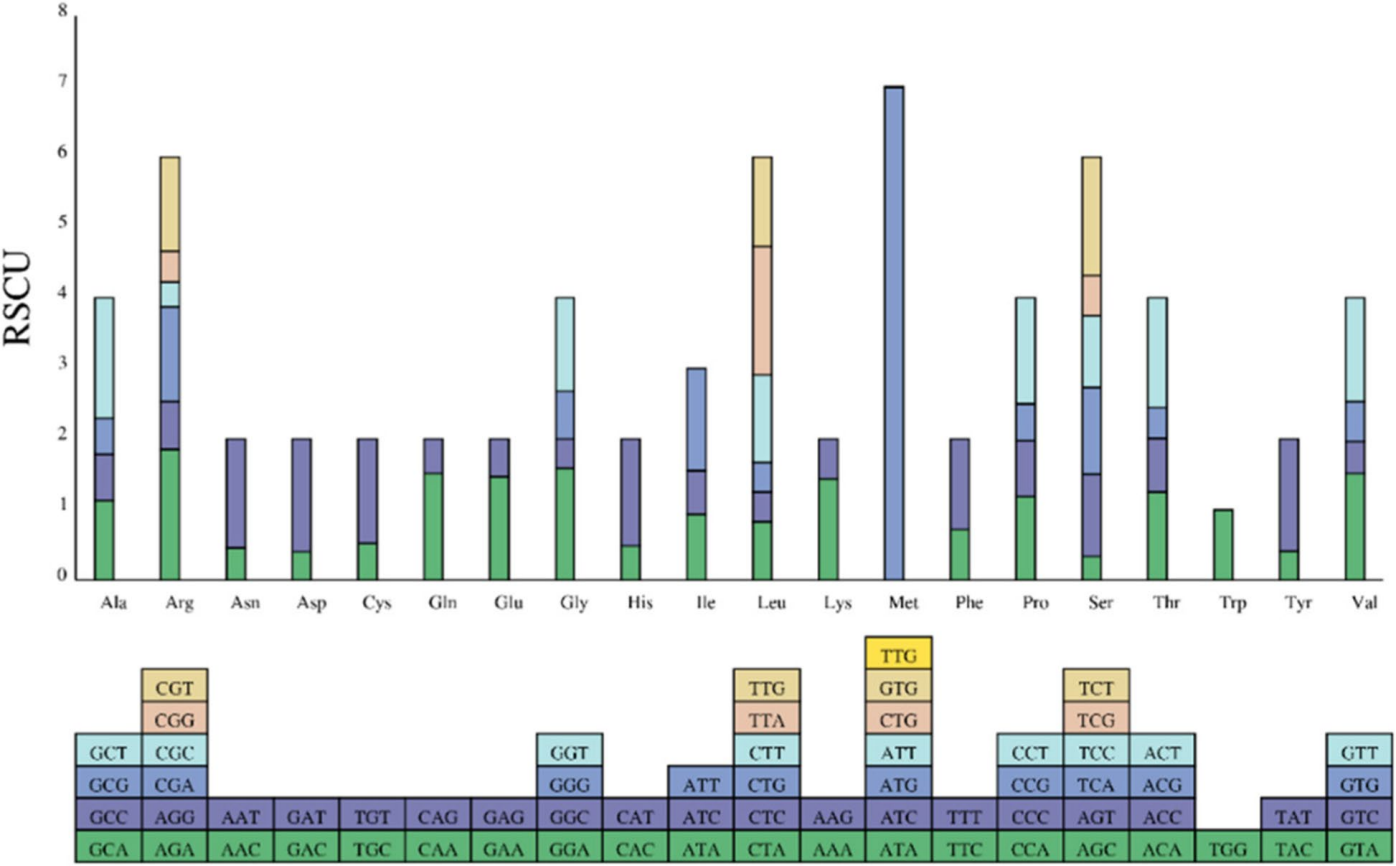
The RSCU of amino acids in A. transsectum's cp genome. The color of the histogram is the same as the codon's color

The codon usage bias (CUB) is a concept that describes to the differential frequency with which numerous synonymous codons encoding the same amino acid are seen [13]. CUB preferences are specific to different genes in different species or even within a particular species, a combination of mutation, selection and drift during the long-term evolution of genes and species [14]. We examined the codon usage frequency of protein-coding genes in the *A. transsectum* cp genome, finding that all proteins were encoded by 26,535 codons (Contains three termination codons) (Table 3 and Fig. 2). Leucine had the most codons (2752 codons, 10.37%), then isoleucine (2243 codons, 8.45%), and serine (2024 codons, 7.63%), while cysteine was the least common amino acid (308 codons, 1.16%). A total of 32 codons (73.4%) had relative synonymous codon usage (RSCU) greater than 1. The most favored codon was AUG, which encodes methionine (Met) and has an RSCU value of 6.9783, followed by AGA, which encodes arginine (Arg) and has an RSCU value of 1.8576.

**Table 3:** Codon list of *A. transsectum*

Codon	AminoAcid	Number	RSCU	Codon	AminoAcid	Number	RSCU
UAA	Ter(*)	39	1.3605	AUU	Met(M)	0	0
UAG	Ter(*)	25	0.8721	CUG	Met(M)	0	0
UGA	Ter(*)	22	0.7674	GUG	Met(M)	2	0.0217
GCA	Ala(A)	395	1.1284	UUG	Met(M)	0	0
GCC	Ala(A)	230	0.6572	AAC	Asn(N)	298	0.4584
GCG	Ala(A)	177	0.5056	AAU	Asn(N)	1002	1.5416
GCU	Ala(A)	598	1.7084	CCA	Pro(P)	341	1.1912
UGC	Cys(C)	81	0.526	CCC	Pro(P)	223	0.7792
UGU	Cys(C)	227	1.474	CCG	Pro(P)	151	0.5276
GAC	Asp(D)	222	0.4018	CCU	Pro(P)	430	1.502
GAU	Asp(D)	883	1.5982	CAA	Gln(Q)	690	1.5132
GAA	Glu(E)	1006	1.4654	CAG	Gln(Q)	222	0.4868
GAG	Glu(E)	367	0.5346	AGA	Arg(R)	501	1.8576
UUC	Phe(F)	531	0.723	AGG	Arg(R)	182	0.675
UUU	Phe(F)	938	1.277	CGA	Arg(R)	362	1.3422
GGA	Gly(G)	725	1.5936	CGC	Arg(R)	95	0.3522
GGC	Gly(G)	187	0.4108	CGG	Arg(R)	118	0.4374
GGG	Gly(G)	306	0.6724	CGU	Arg(R)	360	1.335
GGU	Gly(G)	602	1.3232	AGC	Ser(S)	114	0.3378
CAC	His(H)	166	0.4896	AGU	Ser(S)	394	1.1682
CAU	His(H)	512	1.5104	UCA	Ser(S)	413	1.2246
AUA	Ile(I)	704	0.9417	UCC	Ser(S)	344	1.02
AUC	Ile(I)	459	0.6138	UCG	Ser(S)	193	0.5724
AUU	Ile(I)	1080	1.4445	UCU	Ser(S)	566	1.6776
AAA	Lys(K)	1005	1.444	ACA	Thr(T)	429	1.2508
AAG	Lys(K)	387	0.556	ACC	Thr(T)	260	0.758
CUA	Leu(L)	382	0.8328	ACG	Thr(T)	149	0.4344
CUC	Leu(L)	193	0.4206	ACU	Thr(T)	534	1.5568
CUG	Leu(L)	193	0.4206	GUA	Val(V)	548	1.516
CUU	Leu(L)	566	1.2342	GUC	Val(V)	162	0.448
UUA	Leu(L)	834	1.8186	GUG	Val(V)	205	0.5672
UUG	Leu(L)	584	1.2732	GUU	Val(V)	531	1.4688
AUA	Met(M)	0	0	UGG	Trp(W)	480	1
AUC	Met(M)	0	0	UAC	Tyr(Y)	201	0.4144
AUG	Met(M)	640	6.9783	UAU	Tyr(Y)	769	1.5856

### Interspersed repeats and SSRs

**Fig. 3:**
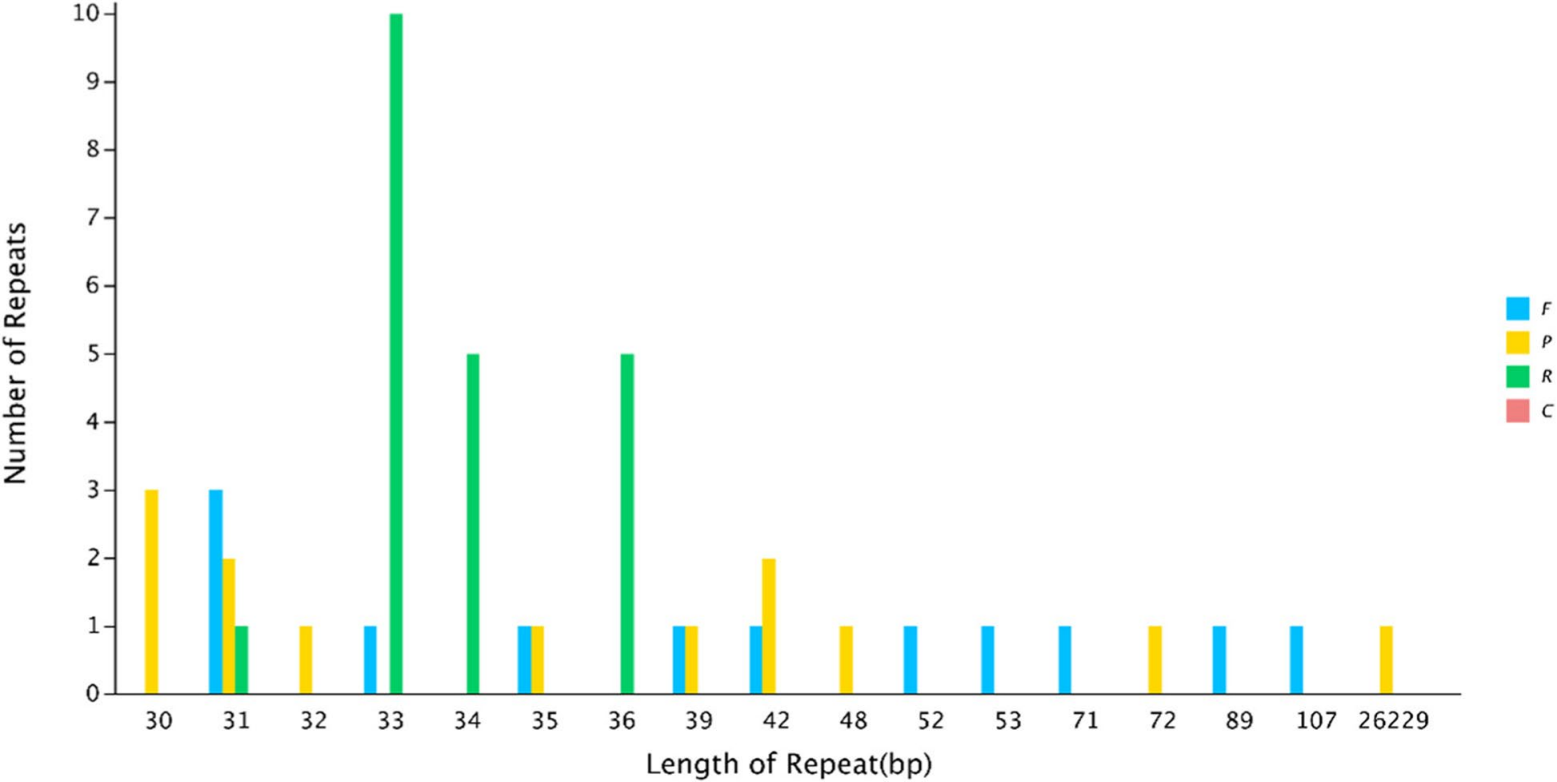
The *A. transsectum* cp genome's repeated sequences. The length of the repetition sequence is the abscissa, and the number of repeat sequences is the ordinate. Forward repetition is abbreviated as F, palindromic repetition is abbreviated as P, reverse repetition is abbreviated as R, and complementary repetition is abbreviated as C

**Fig. 4:**
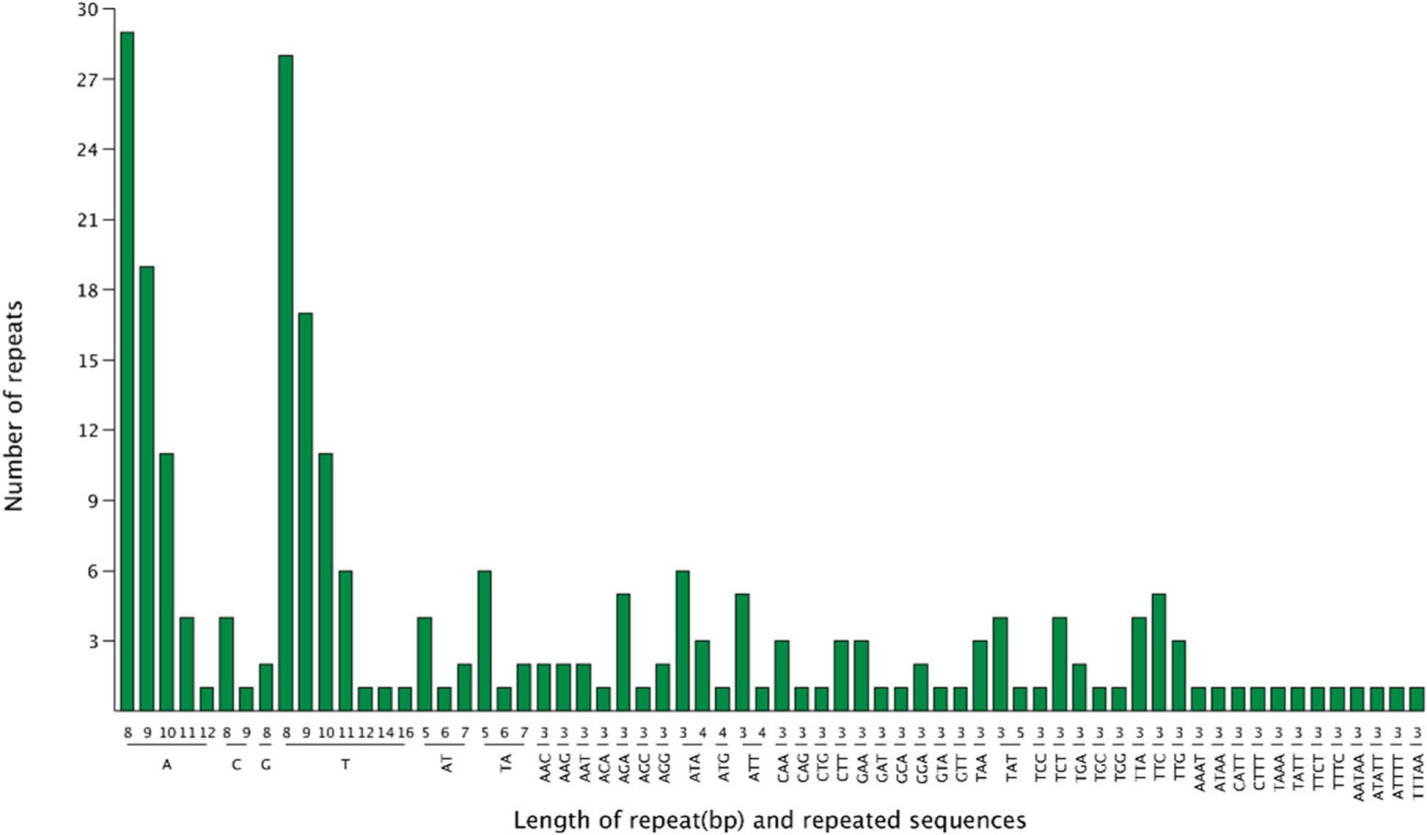
SSRs types in the cp genome of *A. transsectum*. The horizontal axis represents SSR repeats types and the vertical axis is the number of repeats

We discovered 46 interspersed repeats in the *A. transsectum* cp genome, including 21 reverse repeats, 13 palindromic repeats, and 12 forward repeats; complementary repeats were not discovered in the *A. transsectum* cp genome. The length of the repeats ranged from 30 to 26,229 bp, 35 repeats were between 30–39 bp, 10 repeats were between 42–107 bp, and only 1 repeat was 26,229 bp in length (Fig. 3). In the *A. transsectum* cp genome, we found 241 SSRs, 163 of which were found in the LSC region, 40 in the IRs region, and 38 in the SSC region (Fig. 4).

In addition, 137 SSRs were discovered in intergenic spaces, and 104 SSRs were discovered in genes like ycf4, ycf3, ycf2, ycf1, trnI-GAU, trnL-UAA, trnV-UAC, trnK-UUU, rrn23, rps3, rps19, rps18, rps14, rpoC2, rpoC1, rpoB rpoA, rpl22, rpl2, rpl16, rpl14, psbC, psbB, psaJ, psaB, psaA, petA, ndhH, ndhF, ndhD, ndhA, ndhB, matK, accD, ccsA, atpB, atpF, atpI, and other genes. These SSRs consist of 136 mononucleotides, 16 dinucleotides, 77 trinucleotides, 8 tetranucleotides and 4 pentanucleotides. The mononucleotide SSRs were dominated by polyadenine (PolyA) and polythymine (PolyT) repeats (94.86%), with fewer C and G mononucleotides (5.14%).

### Comparative analysis of the cp genomes of A. transsectum and its related species

**Fig. 5:**
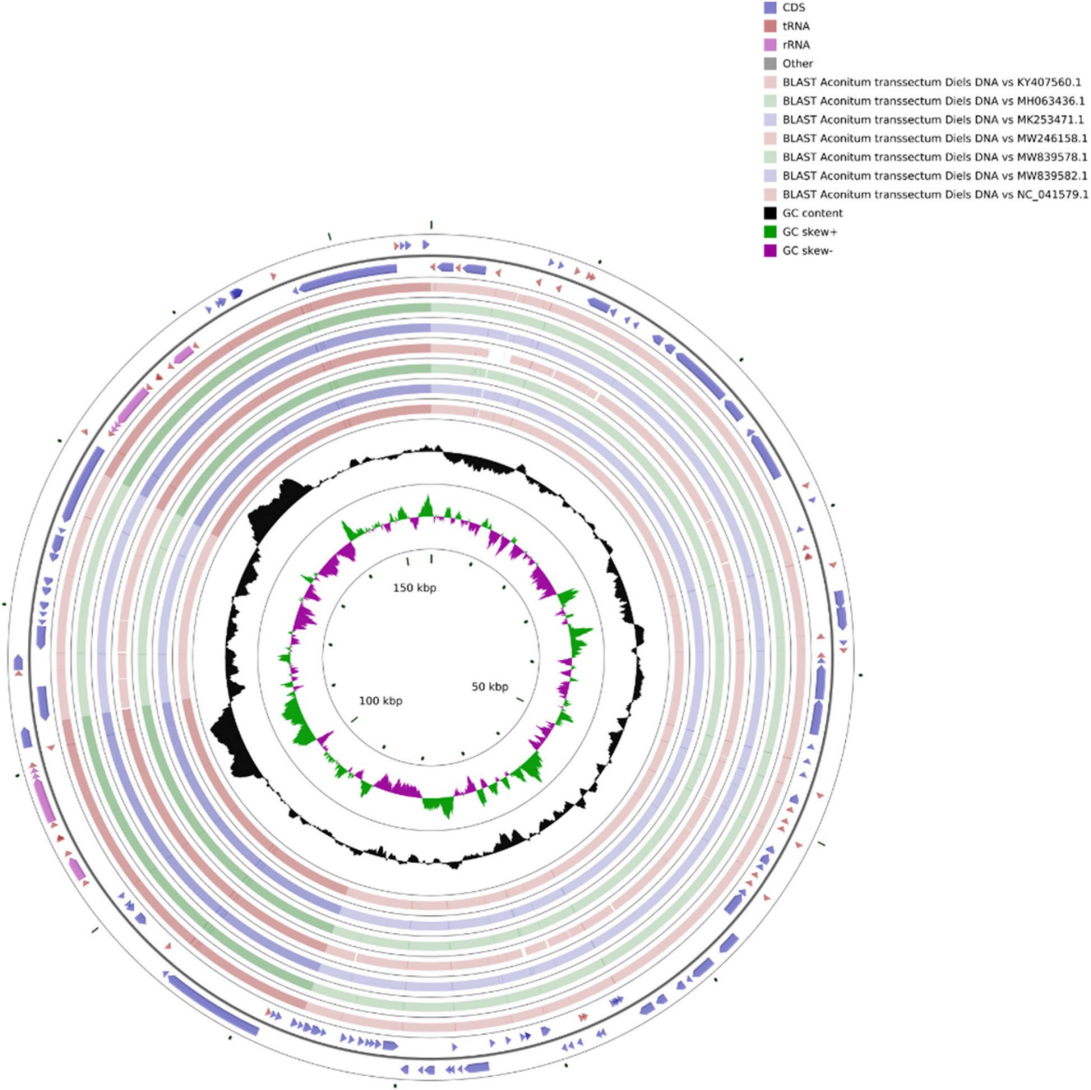
Comparative analysis of cp genome structure. The two outermost circles in the figure represent the genome's gene length and orientation; the seven inner circles represent the similarity results compared to other reference genomes; the black circles represent the GC content; green represents GC-skew + and purple represents GC-skew-; and the black circles represent the GC content

We acquired the cp genome sequences of 7 Ranunculacea species from NCBI to examine the divergence of the *A. transsectum* cp genome from its related specie, including 6 species of the genus *Aconitum* (*A. flavum, A. pendulum, A. brachypodum, A. vilmorinianum, A. kusnezoffii, A. carmichaelii*) and *Delphinium yunnanense*. The results of CGview analysis shown that the cp genomes of *A. transsectum* exhibited high similarity to the 6 species of the genus *Aconite* (Fig. 5). In addition, the cp genomes of *A. transsectum* also similarity to *D. yunnanense,* while it also shown some heterogeneity in LSC and SSC region, as shown by the partial deletion of *D. yunnanense* at 1–20 kbp, 60–90 kbp and 100–120 kbp.

**Fig. 6:**
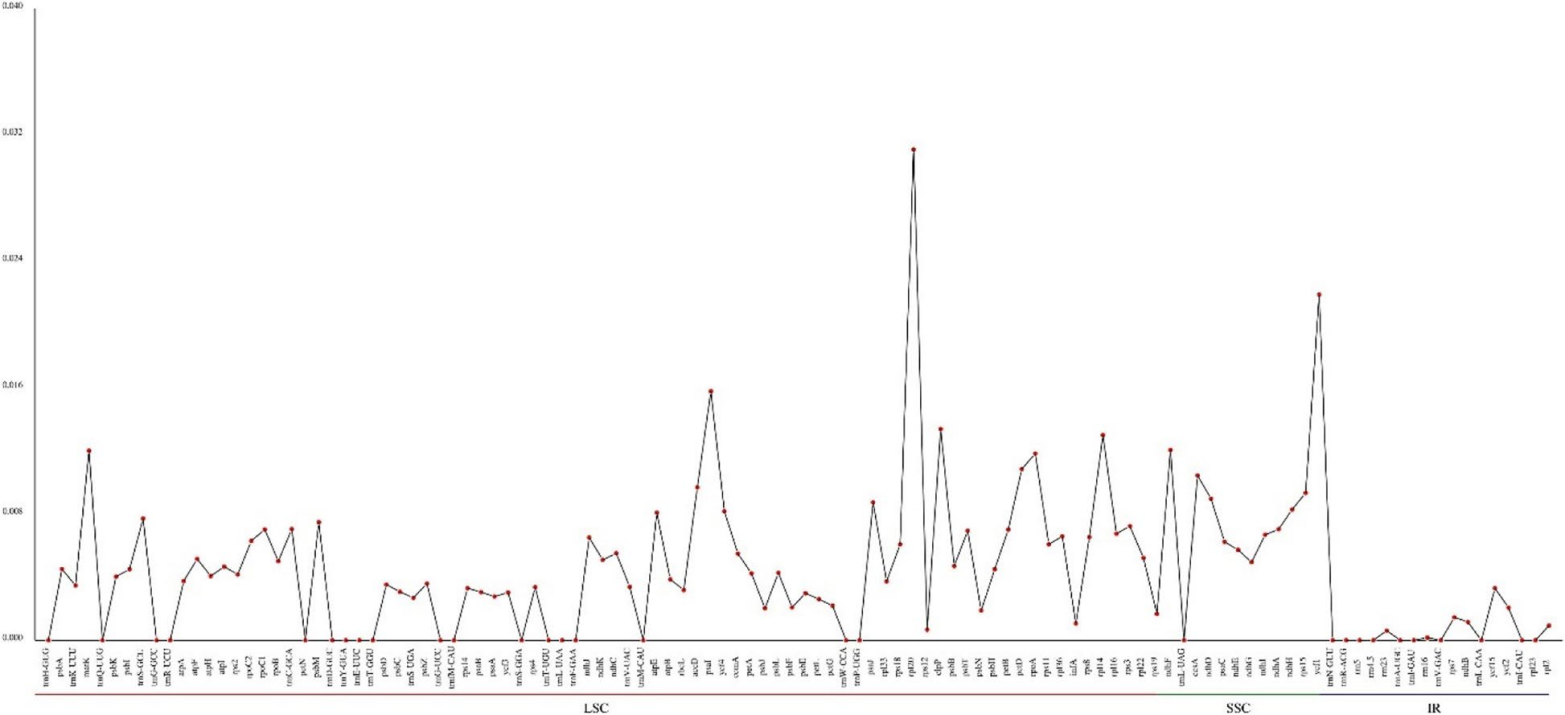
Comparative analysis of Comparative analysis nucleotide diversity. The gene name is indicated by the horizontal coordinate, the PI value is indicated by the vertical coordinate

To inspection the degree of variation in DNA sequences, we examined the nucleotide diversity (PI) values of 112 loci in the cp genome, the PI values of cp genomic sequences ranged from 0–0.03106 with a value of 0.00438 on average (Fig. 6 and Supplementary Table S1). The mean PI value of SSC region was 0.00846, the mean PI value of LSC region was 0.00458, and the mean PI value of IR region was 0.00057, which indicated that the SSC region had the highest nucleotide diversity and the IR region had the lowest nucleotide diversity and was more conserved. In addition, 5 genes with high PI values were detected, including rpl20 (0.03106), ycf1 (0.02187), psaI (0.01577), clpP (0.01338) and rpl14 (0.01299), with rpl20, psaI, clpP and rpl14 in the LSC region and ycf1 in the SSC region. These results suggest that the rpl20, ycf1, psaI, clpP, and rpl14 loci were hypervariable loci (PI > 0.012) at the species level, which could also potentially be developed as barcodes for the identification of *Aconitum*.

**Fig. 7:**
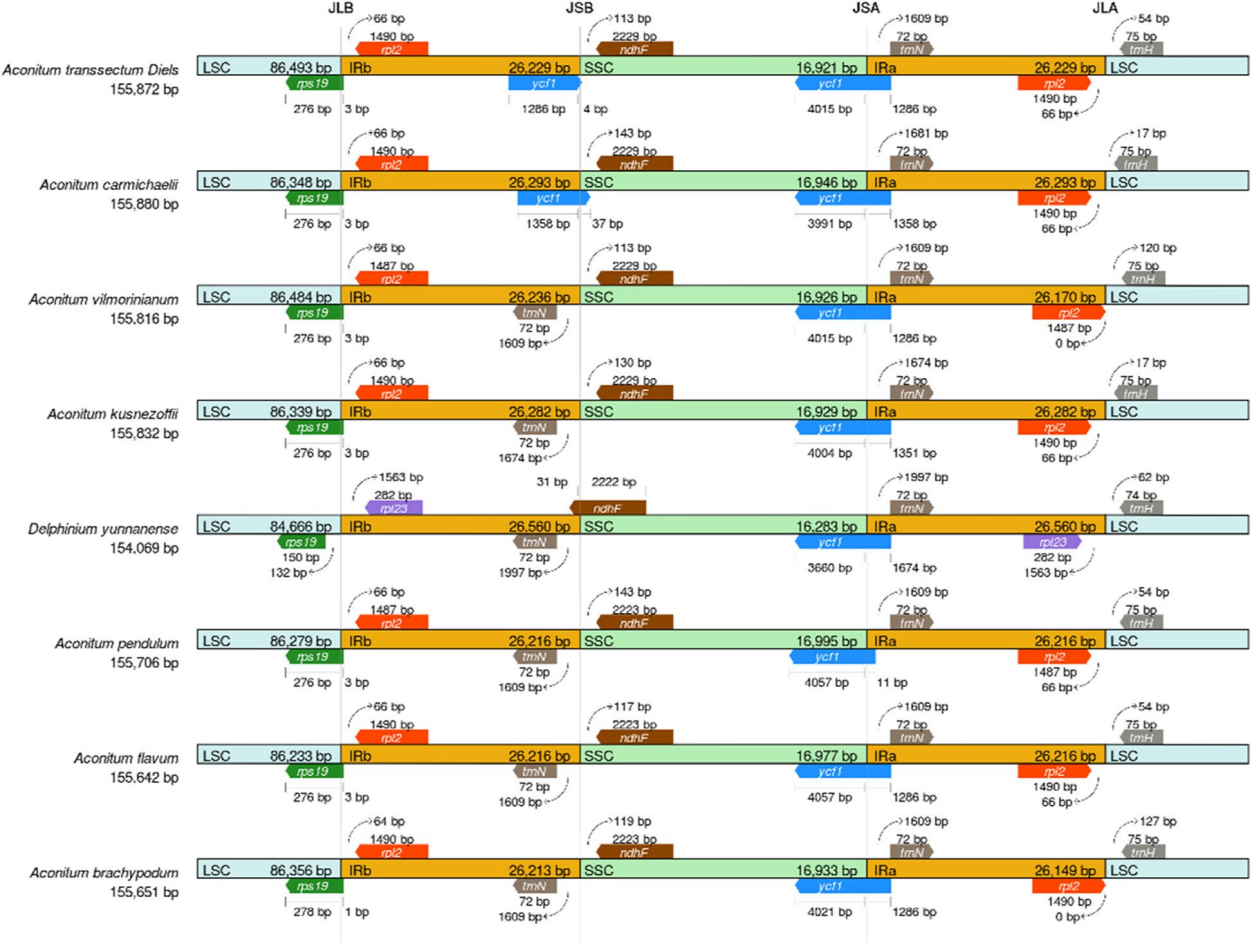
Comparative analysis of IR Expansion and Contraction

As illustrated in Fig. 7, we also investigated at the binding regions of IR/LSC and IR/SSC. The location of the rps19 gene was similar in all 7 *Aconitum* species, spanning the LSC and IRb binding regions, and 1–3 bp distant from the LSC and IRb binding regions, with the exception of *D. yunnanense*, where the rps19 gene was found within the LSC region. The TrnH genes of 7 *Aconitum* species are in the LSC region, 75 bp distant from the IRa/LSC boundary, and only the TrnH gene of *D. yunnanense* is 74 bases away from the IRa/LSC boundary. The ndhF genes of 7 *Aconitum* species are in the SSC region, 113–143 bp distant from the IRa/LSC boundary, and only the ndhF gene of *D. yunnanense* spanning the IRb and SSC binding regions. In addition, the trnN genes of 8 species are located within the IRa region. The above results demonstrated that the cp genome sequences of the 7 *Aconitum* are conserved.

**Fig. 8:**
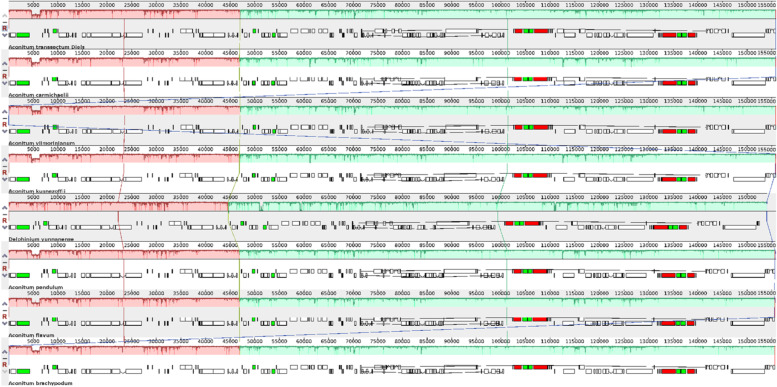
MAUVE alignment of *A. transsectum* related species. As a reference, the cp genome of *A. transsectum* is presented at the top. The long squares show genomic similarity, while the lines connecting them represent a covariate association. Each genome's gene locations are represented by the short squares. CDS is represented by white, tRNA is represented by green, and rRNA is represented by red

The results of the Mauve multiplex alignment analysis show that there are 3 locally collinear blocks (LCB) between the cp genomes of the eight species, indicating a high degree of similarity between the genomes of these 8 species (Fig. 8). Alignment results have shown no rearrangements or inversions between their genomes; however, mutations were observed in regions 5000 to 10,000, characterized by a high degree of gene sequence variation in aligned cp genomes.

**Fig. 9:**
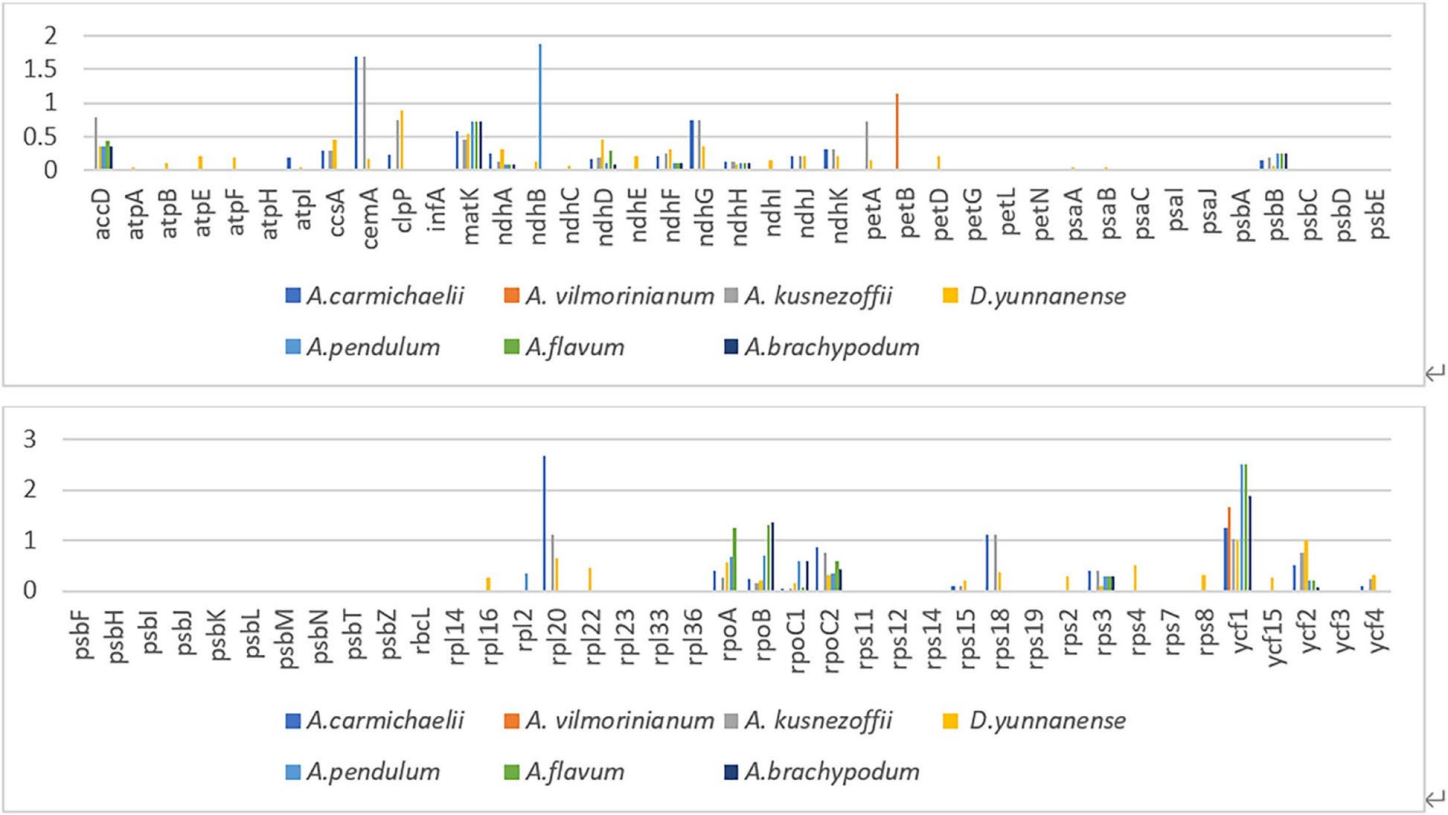
The Ka/Ks analysis was performed on 78 protein-coding genes from the *A.transsectum* cp genome and seven related species

Using *A. transsectum* as a reference, synonymous and nonsynonymous changes in the cp genomes of *A. transsectum* were compared with those of 6 species of the genus *Aconitum* and one species of the genus *Delphinium* were investigated (Fig. 9). The Ka/Ks ratios of 78 protein-coding genes in these 7 cp genomes were found by comparison. The Ka/Ks ratios for the majority of the coding genes were below 1 or could not be determined since either the Ka or Ks values was zero, suggested that they were conserved. The ycf1 gene had Ka/Ks values greater than 1 in all seven species; the rpl20, cemA, and rps18 genes had Ka/Ks values greater than 1 in *A. carmichaelii* and *A. kusnezoffii*; and the rpoB gene had Ka/Ks values greater than 1 in *A. flavum* and *A. brachypodum*.

### Phylogenetic inference

**Fig. 10:**
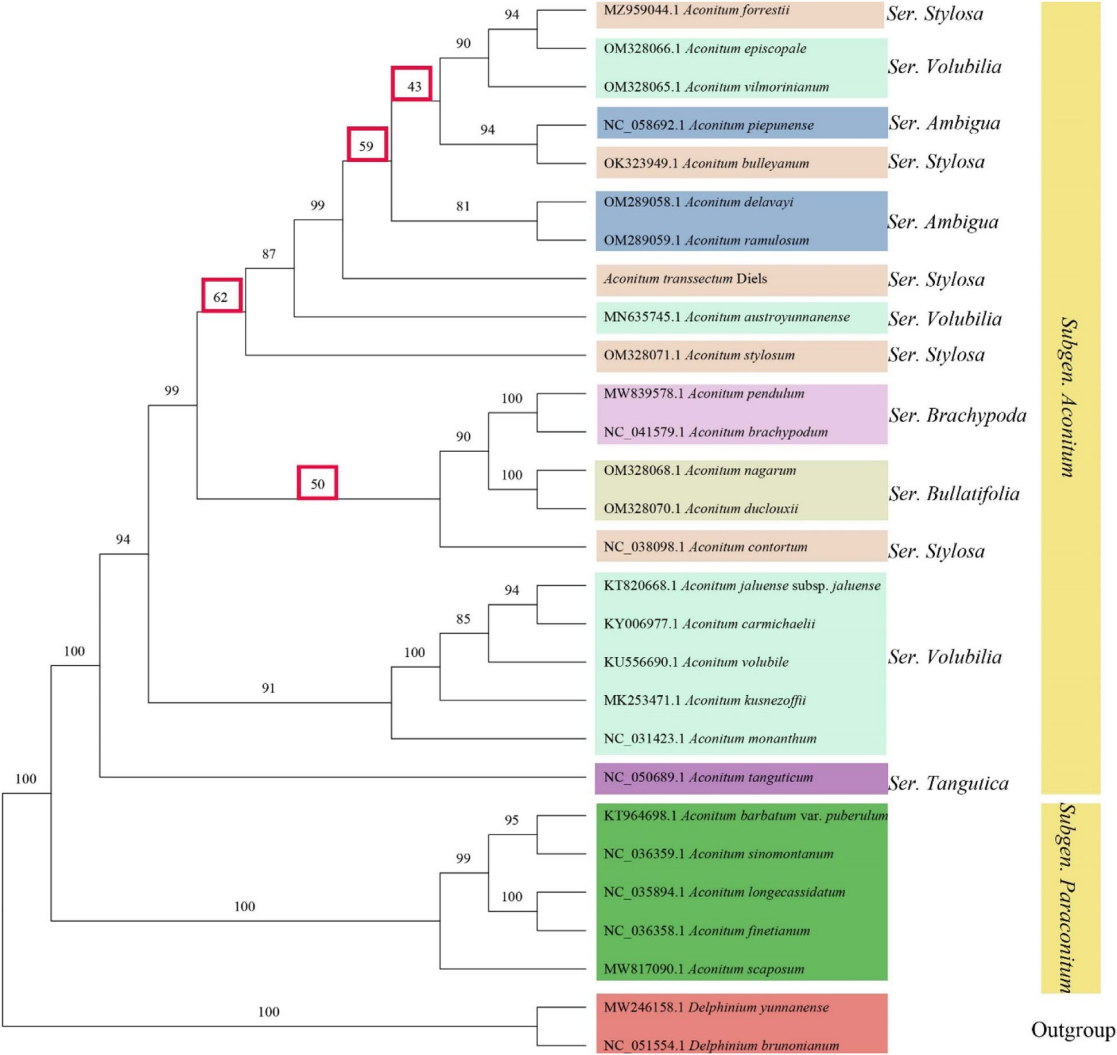
ML phylogenetic tree of 28 species of Ranunculaceae plants constructed with ycf1 gene sequences

Plant phylogenetic studies frequently use cp genomes. The results of comparative analysis of the cp genomes of *A. transsectum* and its related species suggested that ycf1 has potential as a chloroplast DNA barcode for the genus *Aconitum*. Therefore, we utilized the maximum likelihood (ML) method to build a phylogenetic tree of ycf1 gene from 28 species (including 26 species of the genus *Aconitum* and 2 species of the genus *Delphinium*) to Whether ycf1 can be used for phylogenetic analysis within the genus *Aconitum* (Fig. 10). The phylogenetic tree comprised 26 nodes, and the support rate of most nodes was greater than 81 percent (4 nodes was less than 81 percent). The phylogenetic tree separates the subgenera *Delphinium*, *Paraconitum* and *Aconitum* with a very high support rate. However, the branching support rate is low and the classification is confusing when further delineating the subgenus Aconitum.

**Fig. 11:**
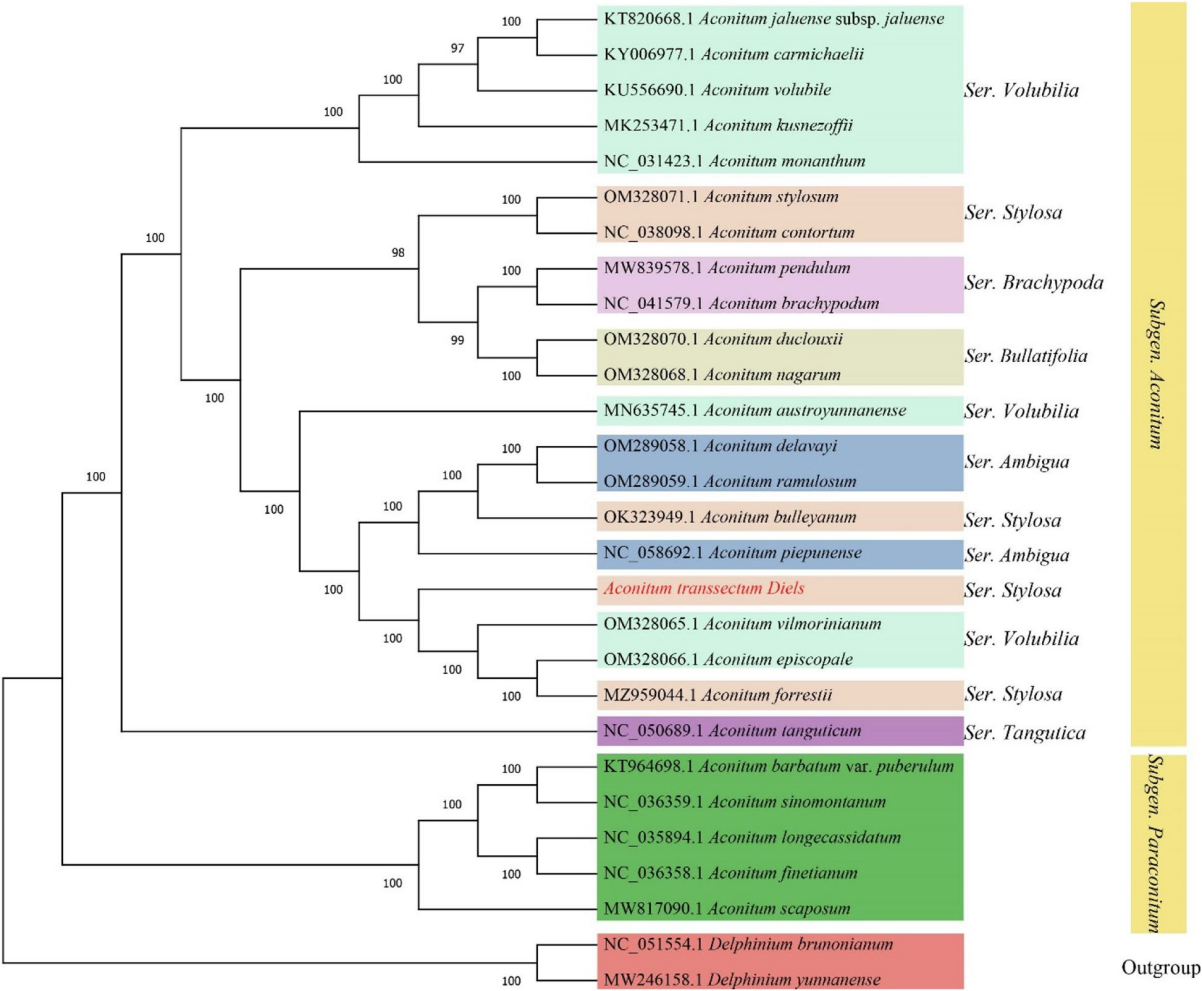
ML phylogenetic tree of 28 species of Ranunculaceae plants constructed with cp genome sequences

After that, we utilized the ML method to build a phylogenetic tree of cp sequences from 28 species (including 26 species of the genus *Aconitum* and 2 species of the genus *Delphinium*) to identify *A. transsectum*'s phylogenetic position (Fig. 11). The phylogenetic tree comprised 25 nodes, and the support rate of all nodes was greater than 97 percent, with 22 nodes having a support rate of 100 percent, indicating that the clustering results were highly reliable. The 28 species might be grouped into three primary taxa on the evolutionary tree. The 21 species of Subgen. *Aconitum* was clustered into one major taxon, the 5 species of Subgen. *paraconitum* were grouped into another major taxon, and the 2 species of the outgroup genus *Delphinium* were grouped into one taxon. *A. transsectum* is located in the subgenus *Aconitum*, and is most closely related to* A. vilmorinianum*, *A. episcopale* and *A. forrestii* of Subgen. *Aconitum*. These results suggest that *A. transsectum* is highly homologous with Subgen. *Aconitum*.

## Discussion

Using Illumina sequence data, we were successful in establishing the complete cp genome sequence of *A. transsectum* in this study. The cp genome of *A. transsectum*, like most land plant cp genomes, has a highly conserved structure and gene content. The size of the *A. transsectum* cp genome is 155872 bp, which is consistent with other members of *Aconitum* (150–157 kb) [15–19]. The *A. transsectum* cp genome is a typical tetrad structure, with four segments (LSC, SSC, and two IR) and highly conserved IR regions. *A. transsectum* is native to northwestern Yunnan and its cp genome size is similar to that of other *Aconitum* species [17], however, the SSC region is longer compared to other species.

Nucleotide diversity (PI) is an indicator that responds to the degree of variation in DNA sequences, and nucleotide diversity responds to the genetic diversity of the species [20]. In chloroplast genes of *A. transsectum* and related species, higher PI values for gene sequences in the LSC/SSC region were observed than in the IR region, which is coherent with other angiosperms [21, 22].

The expansion and contraction of the IR region of the cp genome is a common evolutionary phenomenon [23]. As the genome evolves, there is expansion and contraction of the IR region, when some genes enter the IR region or the LSC and SSC regions [23]. rps19 genes have a tendency to enter the IR region due to the expansion of the LSC/IR boundary in the genus *Aconitum*, while in *D. yunnanense*, the LSC-IR boundary is contracted and the rps19 genes are placed in the LSC region. The above findings indicated that the cp genome boundary genes of the genus *Aconitum* are different from *D. yunnanense*, and those of other genera in the Ranunculaceae family [17, 24].

Repeats and SSRs are widely present in plant cp genomes [25]. Repeats vary in type, number, and location from species to species, and they are used to identify mutational hotspots and phylogenetic links [26]. In this study, we found that *A. transsectum* has 46 repetitive sequences, the number of which is much higher than that of other species in the genus *Aconitum*. Furthermore, the majority of the repeats are found in genes, indicating that the *A. transsectum* cp genome preserves a lot of genetic information. SSR has been frequently utilized to determine phylogenetic relationships, genetic diversity research, and species identification due to their high variability and recessive inheritance [27]. The distribution characteristics of cp SSRs in Cyatheaceae have been shown to be useful for classification among genera [28]. The *A. transsectum* cp genome contained 241 SSRs, the majority of which were identified in the LSC region, which is consistent with observations of cp SSRs in other *Aconitum* species. Further analysis is needed in the future to see whether repeats and SSRs can be used for phylogenetic analysis of the genus *Aconitum*.

A synonymous mutation occurs when a base mutation results in an unmodified amino acid; otherwise, it is a nonsynonymous mutation, and nonsynonymous mutations are frequently affected by natural selection [29]. The counts of nonsynonymous substitutions at each nonsynonymous locus (Ka) and synonymous substitutions at each synonymous locus (Ks) are usually used to indicate the selection effect of a gene (Ks) [30]. When Ka/Ks is more than 1, a positive selection impact is present, and when Ka/Ks is less than 1, a purification selection effect is there [30]. The Ka/Ks of most genes (69 out of 78) were smaller than one in the comparison between *A. transsectum* and the other seven species, indicating that purification selection is essential in these species. In all species, however, the Ka/Ks of ycf1 genes were larger than 1, implying that ycf1 genes were positively selected to adapt to the living environment. The ycf1 gene, the largest chloroplast gene, encodes an ATP-binding cassette (ABC) protein in chloroplasts and generally evolves at a rapid mutation rate [31], as formalized in our study. ycf1 is the most potential chloroplast DNA barcode for land plants since it is very species-specific [32], the ycf1 phylogenetic analysis also shown that ycf1 has taxonomic potential at the subgenus level within the genus *Aconitum*.

Based on the cp genomes of 28 species, an ML phylogenetic tree was created. *A. transsectum* and other species of the subgenus *Aconitum*, such as *A. vilmorinianum, A. episcopale* and *A. forrestii*, constitute a monophyletic branch of the genus *Aconitums*. Phylogeographic results based on morphological features, nuclear DNA markers, and some cp genomes are congruent with our phylogenetic conclusions. Based on ITS sequences, a phylogenetic tree for 51 species of the genus *Aconitum*, including *A. transsectum*, *A. vilmorinianum*, *A. episcopale*, and *A. forrestii*, was previously created [33]. The phylogeny of this ITS sequence showed that *A. transsectum*, *A. vilmorinianum*, *A. episcopale*, and *A. forrestii* are in the same clade and belong to the same subgen. *Aconitum*. Another phylogenetic tree built on 27 cp genomes of *Aconitums* species demonstrates that *A. vilmorinianum*, *A. episcopale*, and *A. forrestii* are all members of the same clade, belonging to the subgen. *Aconitum* [34]. These results clearly reflect the phylogenetic relationships of *A. transsectum* within the genus *Aconitum* and provide reliable evidence for the phylogeny and molecular identification of this traditional medicinal plant.

## Conclusions

In conclusion, the complete cp genome sequence of *A. transsectum* was sequenced and compared to that of other closely related species, providing a crucial reference for *A. transsectum* phylogeny. Although the cp genomes of *A. transsectum* and other *Aconitum* are essentially identical in terms of genome structure, gene content, and gene sequence, the IR region boundary section differs. Because it is exceedingly species-specific, ycf1 is the most promising chloroplast DNA barcode for land plants, and it will give informative markers for phylogenetic research of *Aconitum*. A close relationship has been discovered between *A. transsectum* and *A. vilmorinianum*, *A. episcopale*, and *A. forrestii*, according to phylogenetic research. The findings of this study not only contribute to the creation and utilization of *A. transsectum*, but also serve as a source of reference data for population genomics, phylogenetic analysis, and genetic engineering research.

## Methods

### Ethical statement

For the collection of samples for this study, no special licenses were needed. The relevant Chinese laws were followed as this research was conducted.

### Preparation of materials

The plants were harvested from Machang village, Ludian Town, Yulong County, Lijiang City, and identified as *A. transsectum* by Prof. Su Tai of Yunnan Institute of Materia Medica. For sequencing, fresh young *A. transsectum* leaves were submitted to Genepioneer Biotechnologies in South China.

### DNA extraction, genome sequencing, and annotation

The Cetyl Trimethyl Ammonium Bromide (CTAB) method was used to extract whole genomic DNA from 100 mg of fresh leaves. Paired-end (PE) sequencing with the Illumina NovaSeq 6000 platform, with 150 sequencing read lengths. To screen the raw data and obtain Clean Data of high quality, use the software fastp v 0.20.0. With k-mers of 55, 87, and 121, the cp genome of *A. transsectum* was assembled using SPAdes v3.10.1 [35]. Quality control was performed after assembly using the sequence of *A. piepunense* (accession number NC 058,692.1) [36]. To improve annotation accuracy, we used Prodigal v2.6.3 for cp-coding sequences (CDS), Hmmer v3.1b2 for ribosomal RNA (rRNA) prediction, and Aragorn v1.2.3 for transfer RNA prediction (tRNA). The assembled sequences were then checked using BLAST v2.6 to produce the second annotation results, which were based on sequences of related species that had been published in NCBI. To achieve the final annotation, the two annotation results were manually verified to remove any incorrect or redundant annotations and to establish the exon boundaries. Finally, using the OGDRAW software, the entire genome was mapped [37].

### Codon usage and repeat sequence analysis

Due to codon simplicity, each amino acid has a minimum of 1 codon and a maximum of 6 codons. The genomic codon usage rate differs widely from species to species and organism to organism. Relative Synonymous Codon Usage refers to the inequality in the utilization of synonymous codons (RSCU). This preference is thought to be the outcome of a combination of natural selection, species mutation, and genetic drift. It is computed by dividing the actual codon usage frequency by the theoretical codon usage frequency. The unique CDSs (one copy of the CDS with numerous copies) were filtered using Perl scripts based on the CDSs of the 86 protein-coding genes, and the RSCU of each codon was computed using the software CodonW v1.4.2 [38].

### Comparison of complete Cp genome

The cp genomes of 6 reported *Aconitum* species and 1 exogenous species were loaded from the NCBI website, which are, *A. flavum* (MW839582.1), *A. pendulum* (MW839578.1), *A. brachypodum* (NC_041579.1), *A. vilmorinianum* (MH063436.1), *A. kusnezoffii* (MK253471.1), *A. carmichaelii* (KY407560.1), and *D. yunnanense* (MW246158.1). CGView software was used to evaluate the cp genome structures of the eight plants [39]. Mauve v2.3.1 was used to analyze at the homology and covariance of cp sequences [40]. For broad comparison of homologous gene sequences from different plants, the MAFFT v7.310 (automatic mode) [41] was employed. Nucleotide diversity (PI) values for each gene were calculated using DNAsp v5.0 [42]. The IR, SSC, and LSC region boundary information was visualized using the SVG package in Perl. MAFFT v7.310 software was used to compare gene sequences, and Ka/Ks Calculator v2.0 software was utilized to calculate the Ka/Ks values of the genes.

### Phylogenetic evaluation

Additional cp genome sequences and ycf1 gene sequences were obtained from the NCBI website for 25 *Aconitum* species and 2 *Delphinium* specie, which are, *A. pendulum* (MW839578.1), *A. brachypodum* (NC_041579.1), *A. vilmorinianum* (OM328065.1), *A. kusnezoffii* (MK253471.1), *A. carmichaelii* (KY006977.1), *A. longecassidatum* (NC_035894.1), *A. piepunense* (NC_058692.1), *A. scaposum* (MW817090.1), *A. bulleyanum* (OK323949.1), *A. austroyunnanense* (MN635745.1), *A. tanguticum* (NC_050689.1), *A. episcopale* (OM328066.1), *A. delavayi* (OM289058.1), *A. contortum* (NC_038098.1), *A. sinomontanum* (NC_036359.1), *A. finetianum* (NC_036358.1), *A. volubile* (KU556690.1), *A. barbatum* var*. puberulum* (KT964698.1), *A. monanthum* (NC_031423.1), *A. jaluense* subsp. *jaluense* (KT820668.1), *A. stylosum* (OM328071.1), *A. duclouxii* (OM328070.1), *A. nagarum* (OM328068.1), *A. ramulosum* (OM289059.1), *A. forrestii* (MZ959044.1), *D. yunnanense* (MW246158.1)*, and D. brunonianum* (NC_051554.1). The sequence alignment was conducted by MAFFT [43] based on the cp genome sequences of 28 species, including *A. transsectum*; the alignment results were further optimized by trimAl software [44]. The maximum likelihood (ML) phylogenetic tree was constructed with IQ-TREE 1.6.12 [45] using *D. yunnanense* and *D. brunonianum* as outgroups, with Bootstrap value set to 1000, and the best tree building module was selected by the built-in Model Finder of IQ-TREE based on the optimized alignment result.

## Supplementary Information

**Additional file 1.**

## Data Availability

The datasets generated and analyzed in this study are available in the GenBank of NCBI, and the complete chloroplast genome sequence of *Aconitum transsectum* is deposited in GenBank of NCBI under accession number ON751949.1. The accession numbers for the remaining datasets used and analyzed in this study are listed in the Methods section.

## Abbreviations

A.transsectum: Aconitum transsectum Diels
cp: Chloroplast
LSC: Large single copy
SSC: Small single copy
IR: Inverted repeat
CUB: Codon usage bias
RSCU: Relative synonymous codon usage
Met: Methionine
Arg: Arginine
PolyA: Polyadenine
PolyT: Polythymine
LCB: Locally collinear blocks
ML: Maximum likelihood
PI: Nucleotide diversity
Ka: Nonsynonymous locus
Ks: Synonymous locus
ABC: ATP-binding cassette
CTAB: Cetyl Trimethyl Ammonium Bromide
PE: Paired-end
CDS: Cp-coding sequences
rRNA: Ribosomal RNA
tRNA: Transfer RNA
ML: Maximum likelihood
